# Assessment of syndromic management of curable sexually transmitted and reproductive tract infections among pregnant women: an observational cross-sectional study

**DOI:** 10.1186/s12884-021-03573-3

**Published:** 2021-01-30

**Authors:** Enesia Banda Chaponda, Jane Bruce, Charles Michelo, Daniel Chandramohan, R. Matthew Chico

**Affiliations:** 1 Department of Biological Sciences, University of Zambia, Lusaka, Zambia; 2 Department of Disease Control, Faculty of Infectious and Tropical Diseases, London School of Hygiene and Tropical Medicine, London, UK; 3 Department of Epidemiology, School of Public Health, University of Zambia, Lusaka, Zambia; 4 Strategic Centre for Health Systems Metrics and Evaluations, School of Public Health, University of Zambia, Lusaka, Zambia

**Keywords:** Syndromic management, Sexually transmitted infections, Reproductive tract infections, Bacterial vaginosis, Prevalence, Risk factors, Sub-Saharan Africa

## Abstract

**Background:**

This study estimated the prevalence of curable sexually transmitted and reproductive tract infections (STIs/RTIs) among pregnant women attending antenatal care (ANC) in rural Zambia, evaluated the effectiveness of syndromic management of STIs/RTIs versus reference-standard laboratory diagnoses, and identified determinants of curable STIs/RTIs during pregnancy.

**Methods:**

A total of 1086 pregnant women were enrolled at ANC booking, socio-demographic information and biological samples were collected, and the provision of syndromic management based care was documented. The Piot-Fransen model was used to evaluate the effectiveness of syndromic management versus etiological testing, and univariate and multivariate logistic regression analyses were used to identify determinants of STIs/RTIs.

**Results:**

Participants had a mean age of 25.6 years and a mean gestational age of 22.0 weeks. Of 1084 women, 700 had at least one STI/RTI (64.6%; 95% confidence interval [CI], 61.7, 67.4). Only 10.2% of infected women received any treatment for a curable STI/RTI (excluding syphilis). Treatment was given to 0 of 56 women with chlamydia (prevalence 5.2%; 95% CI, 4.0, 6.6), 14.7% of participants with gonorrhoea (prevalence 3.1%; 95% CI, 2.2, 4.4), 7.8% of trichomoniasis positives (prevalence 24.8%; 95% CI, 22.3, 27.5) and 7.5% of women with bacterial vaginosis (prevalence 48.7%; 95% CI, 45.2, 51.2). An estimated 7.1% (95% CI, 5.6, 8.7) of participants had syphilis and received treatment. Women < 20 years old were more likely (adjusted odds ratio [aOR] = 5.01; 95% CI: 1.23, 19.44) to have gonorrhoea compared to women ≥30. The odds of trichomoniasis infection were highest among primigravidae (aOR = 2.40; 95% CI: 1.69, 3.40), decreasing with each subsequent pregnancy. Women 20 to 29 years old were more likely to be diagnosed with bacterial vaginosis compared to women ≥30 (aOR = 1.58; 95% CI: 1.19, 2.10). Women aged 20 to 29 and ≥ 30 years had higher odds of infection with syphilis, aOR = 3.96; 95% CI: 1.40, 11.20 and aOR = 3.29; 95% CI: 1.11, 9.74 respectively, compared to women under 20.

**Conclusions:**

Curable STIs/RTIs were common and the majority of cases were undetected and untreated. Alternative approaches are urgently needed in the ANC setting in rural Zambia.

**Supplementary Information:**

The online version contains supplementary material available at 10.1186/s12884-021-03573-3.

## Background

Curable sexually transmitted and reproductive tract infections (STIs/RTIs) – chlamydia, gonorrhoea, trichomoniasis, bacterial vaginosis, and syphilis – are important causes of adverse pregnancy outcomes. Chlamydia (*Chlamydia trachomatis*) increases the odds of preterm birth four-fold [1], and more than doubles the chances of experiencing premature rupture of the membranes [1], delivering a newborn who is low birthweight [1] or small-for-gestational age [2]. Gonorrhoea (*Neisseria gonorrhoeae*) is associated with a three-fold increase in preterm birth [3]. Trichomoniasis (*Trichomonas vaginalis*), the most prevalent curable STI in the world, increases the odds of preterm delivery 1.5 times [4]. Bacterial vaginosis, the most common urogenital disorder in the world among women of reproductive age, increases the odds of preterm delivery two-fold [4]. Bacterial vaginosis and trichomoniasis both double the odds of delivering a low birthweight child [4]. Syphilis (*Treponema pallidum*) is associated with four of every ten spontaneous abortions [5, 6], and one-quarter of stillbirths [7]. Pregnant women who have curable STIs are at elevated risk of HIV acquisition during pregnancy [8, 9], whereas HIV-infected pregnant women who have curable STIs at delivery are at increased risk of giving birth to an HIV-infected infant [10].

To reduce the burden of congenital syphilis, the World Health Organization (WHO) recommends universal screening of pregnant women at antenatal care (ANC) booking and the provision of 2.4MU benzathine penicillin G to individuals who test positive. For the diagnosis of urethral discharge and genital ulcer disease that include chlamydia, gonorrhoea, trichomoniasis, and bacterial vaginosis, the WHO recommends the use of diagnostic and treatment algorithms called syndromic case management [11] in low-resource areas where laboratory facilities and trained staff are unavailable. Syndromic management is based on the identification of consistent groups of symptoms and easily recognized signs and treatment that is effective against the majority or most serious organisms responsible for producing a syndrome. This approach can be effective for diagnosing and treating men. However, curable STIs/RTIs are often asymptomatic among women for whom the algorithms fail to diagnose 70 to 80% of chlamydial and gonococcal infections [12], and 60 to 70% of trichomoniasis and bacterial vaginosis cases [13, 14].

The co-infection prevalence estimates of STI/RTI and malaria infection among pregnant women in the Nchelenge District of Zambia has been previously reported [15]. Nchelenge is a rural area with a population of approximately 173,680 [16] on the shores of Lake Mweru in the Luapula Province. In this analysis the prevalence of curable STIs/RTIs among pregnant women, effectiveness of the syndromic case management for diagnosis of curable STIs/RTIs compared to reference-standard laboratory assays, and risk factors for having a curable STI/RTI during pregnancy are presented.

## Methods

The main study involved an observational cohort of women enrolled at first ANC visit and followed to delivery. Participants were enrolled (*n* = 1086) who were less than 32 gestational weeks measured by ultra-sound during their first ANC visit to Nchelenge and Kashikishi health facilities from November 2013 to April 2014 [17].

Consistent with Zambian ANC policy, clinic staff screened pregnant women and their partners for HIV [18, 19]. Venous blood was collected from women for syphilis testing by rapid plasma reagin (RPR) (Omega Diagnostics Limited, Alva, Scotland, United Kingdom) and RPR-positive women were treated with 2.4 MU of benzathine penicillin G together with their partners according to national norms [20]. Clinic staff recorded participant socio-demographic information and obstetric history during the first visit. As part of standard ANC, clinic staff asked women at antenatal care visits if they had experienced any symptoms associated with STIs/RTIs prior to enrolment. If women responded in the affirmative, they were screened further and treated based on syndromic management guidelines by the clinic staff. Apart from routine care, clinic staff collected cervico-vaginal swabs and stored them at the district hospital. Samples were transported to the University Teaching Hospital in Lusaka where molecular detection of the presence of each organism of interest, *C. trachomatis*, *N. gonorrhoeae* and *T. vaginalis* was conducted. Polymerase chain reaction methods were used to identify species of interest with positive and negative controls at the stages of extraction, amplification and electrophoresis [21–23]. Bacterial vaginosis was diagnosed by microscopy using gold standard Nugent criteria [24]. Results of these assays were communicated to participating clinics and study staff who traced positive cases to their homes and encouraged women to seek treatment from the clinics at no cost. Separately, to confirm syphilis positivity, *Treponema* *pallidum* haemagglutination assays were performed (Chronolab Systems, Barcelona, Spain) on samples that were RPR seropositive. 

### Data processing and analysis

Data were double-entered and cleaned in EpiData software version 3.1 [25] and analysed with Stata IC 13 software [26]. The prevalence of curable STIs/RTIs and 95% CIs were estimated, and the Piot-Fransen model was applied to evaluate the effectiveness of syndromic case management versus etiological testing in the ANC setting. The Piot-Fransen model has been previously used to identify weaknesses in case management of STIs [27], malaria [28], and tuberculosis [29]. The prevalence estimates were stratified by HIV status and odds ratios (OR) were estimated in univariate logistic regression models that contained variables we considered a priori to be potential risk factors. For each infection, risk factors were then assessed in a multivariable model using a likelihood-ratio test. Risk factors that were assessed included maternal age, gravidity, marital status, years of schooling, number of life-time sexual partners, self-reported STI/RTI related symptoms and HIV status. The final model for each infection included risk factors that demonstrated an effect in the adjusted model at the 5% significance level and on the adjusted OR estimates for individual STIs/RTIs. Models were also produced for having any STI/RTI.

## Results

In total, 1086 women were recruited to the study, with one withdrawing consent during the study. Background characteristics of participants have been reported elsewhere [15]. Briefly, women had a mean age of 25.6 years (95% CI: 25.1, 25.9) and a mean gestational age of 22.0 weeks (95% CI: 21.7, 22.2); 55.3% (600 of 1085) had some primary school education, and 80.6% (874 of 1085) were married. The mean age of sexual debut was 17.2 years (range 10 to 28 years). Overall, 13.2% (95% CI: 11.3, 15.3) of women tested positive for HIV. Multigravidae (99 of 658) had the highest prevalence of HIV, 15.0% (95% CI: 12.5, 18.0), followed by secundigravidae (23 of 165) at 13.9% (95% CI: 9.4, 20.1%), and 8.0% (95% CI: 5.3, 12.0%) among primigravidae (21 of 261), *P*-value = 0.018.

### Prevalence of individual STIs/RTIs

In total, samples from 1084 women were available for analysis. However, three samples for bacterial vaginosis and seven samples for syphilis were not evaluable. Table 1 contains a complete summary of prevalence estimates of STIs/RTIs for all women and those who were HIV positive. Among women tested, 64.6% (95% CI, 61.7, 67.4) had at least one curable STI/RTI (700 of 1084). An estimated 5.2% (95% CI: 3.9, 6.7) of participants had chlamydia (56 of 1084) with no significant difference between women with HIV and those without. Among the five curable STIs/RTIs investigated, gonorrhoea was the least prevalent (34 of 1084) at 3.1% (95% CI: 2.2, 4.4). Gonorrhoea was more common among HIV-infected women compared to those without HIV. One-quarter of women (269 of 1084) had trichomoniasis, 24.8% (95% CI: 22.3, 27.5), and there was no difference in the prevalence between HIV-infected and HIV-uninfected women. Bacterial vaginosis was diagnosed among 48.2% (95% CI: 45.2, 51.2) of women (521 of 1081). Participants with HIV were more likely to have bacterial vaginosis, than HIV-uninfected women. An estimated 7.1% (95% CI: 5.6, 8.7) of participants had syphilis (76 of 1077) and the proportion was more than double among women with HIV.

**Table 1:** Prevalence of curable STIs/RTIs among all pregnant women (*N* = 1084) at antenatal care booking and by HIV status

Curable STI/RTI (n)	Cases among all women		Cases among HIV-infected women*		
	% positive	95% CI	% positive	95% CI	***P***-value^**¥**^
**Chlamydia**					
Positive	56 (5.2)	3.9, 6.7	10 (7.0)	3.8, 12.6	0.291
Paucigravidae (426)	28 (6.6)	4.6, 9.4	5 (11.4)	4.7, 24.5	0.236
Multigravidae (658)	28 (4.3)	3.0, 6.1	5 (5.1)	2.1, 11.7	0.718
**Gonorrhoea**					
Positive	34 (3.1)	2.2, 4.4	9 (6.3)	3.3, 11.7	0.020
Paucigravidae (426	17 (4.0)	2.5,6.3	3 (6.8)	2.2, 19.5	0.227
Multigravidae (658)	17 (2.6)	1.6, 4.1	6 (6.1)	2.7, 12.9	0.059
**Trichomoniasis**					
Positive	269 (24.8)	22.3, 27.5	34 (23.8)	17.5, 31.5	0.752
Paucigravidae (426)	139 (32.6)	28.3, 37.2	13 (29.5)	17.8, 44.8	0.676
Multigravidae (658)	130 (19.8)	16.9, 23.0	21 (21.2)	14.2, 30.5	0.745
**Bacterial vaginosis**					
Positive	521 (48.2)	45.2, 51.2	95 (66.4)	58.3, 73.7	< 0.001
Paucigravidae (423)	212 (50.1)	45.4, 54.9	31 (70.5)	55.2, 82.2,	0.001
Multigravidae (658)	309 (47.0)	43.2, 50.7	64 (64.6)	55.6, 73.5	0.001
**Syphilis**					
Positive	76 (7.1)	5.6, 8.7	22 (15.8)	10.6, 23.0	< 0.001
Paucigravidae (425)	28 (6.6)	4.5, 9.3	11 (25.6)	14.6, 40.9	< 0.001
Multigravidae (652)	48 (7.4)	5.6, 9.6	11 (11.5)	6.4, 19.7	0.160
**Any STI/RTI**					
Positive	700 (64.6)	61.7, 67.4	110 (76.9)	69.2, 83.2	0.001
Paucigravidae (426)	294 (69.0)	64.4, 73.2	38 (86.4)	72.5, 93.8	0.016
Multigravidae (658)	406 (61.7)	57.9, 65.3	72 (72.7)	63.0, 80.7	0.034

*HIV* Human Immunodeficiency Virus

Samples that were processed for the detection of each infection were as follow; detection of chlamydia, gonorrhoea and trichomoniasis 1084 samples instead of 1085 were processed; detection of syphilis 1077 instead of 1085 and for detection of bacterial vaginosis 1081 instead of 1085 samples were processed due to missing samples

The HIV status of one participant was not determined and, therefore, was excluded from this analysis

*A total of 143 participants were HIV positive, 44 paucigravidae and 99 multigravidae

^**¥**^ *P*-value obtained from the two-sample test of proportions which was used to test the difference in prevalence of each infection among HIV positive and HIV negative women

### Effectiveness of syndromic case management

**Fig. 1:**
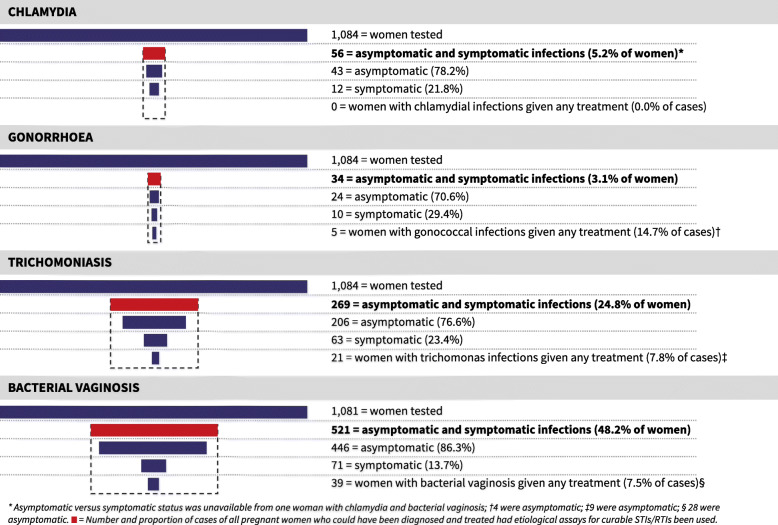
Effectiveness of syndromic management of curable STIs/RTIs among pregnant women in the Nchelenge District, Zambia. * Asymptomatic versus symptomatic status was unavailable from one woman with chlamydia and bacterial vaginosis; †4 were asymptomatic; ‡9 were asymptomatic; § 28 were asymptomatic

Overall, 12.4% of women self-reported symptoms associated with STIs/RTIs at the time of recruitment and 18.0% reported experiencing symptoms during the pregnancy duration including at recruitment. Of women who had at least one curable STI/RTI, 86.0%, (602 of 700) were asymptomatic at recruitment. Among women with chlamydia, 83.9% (47 of 56) were asymptomatic, as were 76.5% (26 of 34) of gonorrhoea cases, 84.4% (227 of 269) of trichomoniasis cases, and 86.0% (448 of 521) of women diagnosed with bacterial vaginosis. Figure 1 illustrates the Piot-Fransen model and quantifies the number as well as proportion of women tested for each STI/RTI, the proportion of symptomatic versus asymptomatic infections identified, and the number of women with STIs/RTIs who received treatment. Overall, only 10.2% (69 of 680) of curable STI/RTI cases, excluding syphilis, received treatment. Of the 98 symptomatic women, 43 cases were given treatment. Not one chlamydial infection of 56 was treated. In contrast, treatment was provided to 14.7% of women with gonococcal infections (5 of 34), 7.8% of trichomoniasis cases (21 of 269), and 7.5% of women with bacterial vaginosis (39 of 521).

The proportion of women experiencing symptomatic STIs/RTIs at recruitment was higher among HIV-positive women, 19.6%, (28 of 143) compared to HIV-negative women, 11.3%, (106 of 941), *P*-value = 0.005. More HIV-positive participants received treatment than HIV-negative women, but this was not statistically significant, 16.4% versus 10.5%, respectively, *P*-value = 0.076.

### Risk factors for STIs/RTIs

Among the five curable STIs/RTIs, only chlamydia had no particular risk factor that was identified to be associated with infection. HIV infection was associated with having gonorrhoea, (aOR = 2.80; 95% CI: 1.25, 6.26), adjusted for age; bacterial vaginosis (aOR = 2.41; 95% CI: 1.66, 3.51), adjusted for age; syphilis infection (aOR = 2.56; 95% CI: 1.48, 4.41) adjusted for age; the number of life-time sexual partners and having any STI/RTI (aOR = 2.10; 95% CI: 1.39, 3.19), adjusted for age.

There was an inverse association between age and gonorrhoea. Compared to women 30 years and older, the odds of gonococcal infection were five-times greater among pregnant women under 20 years of age (aOR = 5.01; 95% CI: 1.29,19.44), and four-times higher among participants between 20 and 29 (aOR = 4.27; 95% CI: 1.32, 14.94) after adjusting for HIV infection. A similar relationship between gravidity and trichomoniasis was identified. In contrast to multigravidae (4th), primigravidae had the highest odds of trichomonas infection (aOR = 2.40; 95% CI: 1.69, 3.40), followed by secundigravidae (aOR = 2.06; 95% CI: 1.37, 3.10), and then multigravidae (3rd) (aOR = 1.58; 95% CI: 1.05, 2.39) after adjusting for self-reported STI/RTI symptoms. Women 20 to 29 years of age had a 58% greater chance of experiencing bacterial vaginosis (aOR = 1.58; 95% CI: 1.19, 2.10) compared to women 30 years of age and older adjusted for HIV infection. Women aged below 20 years had higher odds of being diagnosed with bacterial vaginosis compared to women aged 30 years and older, but this association was not statistically significant. However, compared to women aged 30 years and above, women aged 20–29 had increased odds of infection with bacterial vaginosis, aOR = 1.58; 95% CI: 1.19, 2.10 after adjusting for HIV status. Women younger than 20 years of age, and those between 20 to 29 years, had higher odds of having any STI/RTI compared to pregnant counterparts 30 years and above, aOR = 1.70; 95% CI: 1.16, 2.78 and aOR = 1.58; 95% CI: 1.18, 2.11, respectively, after adjusting for HIV infection.

There was a statistically significant association between syphilis and age. Compared to participants under the age of 20, the odds of a syphilis infection were four-times higher among women from 20 to 29, (aOR = 3.96;95% CI: 1.40, 11.20), and three-times higher among women 30 and older, (aOR = 3.29; 95% CI: 1.11, 9.74) adjusted for HIV infection and number of life-time sexual partners. The odds of having syphilis nearly doubled among women who had three or more life-time partners versus those who had just one or two partners (aOR = 1.82; 95% CI: 1.12, 2.98) adjusted for HIV-infection and age. Table 2 shows a summary of the crude and adjusted ORs of risk factors for each STI/RTI. Supplementary Tables 1, 2, 3, 4, 5, 6 contain complete results for the risk-factor models for individual infections and composite STIs/RTIs.

**Table 2:** Risk factors associated with gonorrhoea, trichomoniasis, syphilis, bacterial vaginosis and any sexually transmitted and reproductive tract infection among pregnant women at antenatal care booking

Risk factor	% positive	% negative^**¥**^	Women per strata	Crude odds ratio (95% CI)	Adjusted odds ratio^*^ (95% CI)
**Gonorrhoea**					
**Age group**					
< 20	3.9	96.1	204	4.14 (1.08, 15.78)	**5.01 (1.29, 19.44)**
20–29	4.0	96.0	573	4.24 (1.26, 14.22)	**4.27 (1.32, 14.94)**
30+	1.0	99.0	307	Ref	Ref
**HIV status**					
Negative	2.7	97.3	940	Ref	Ref
Positive	6.3	93.7	143	2.46 (1.12, 5.38)	**2.80 (1.25, 6.26)**
**Trichomoniasis**					
**Pregnancy number**					
Primigravidae	34.1	65.9	261	2.39 (1.69, 3.38)	**2.40 (1.69, 3.40)**
Secundigravidae	30.3	69.7	165	2.01 (1.34, 3.02)	**2.06 (1.37, 3.10)**
Multigravidae (3rd)	25.0	75.0	180	1.54 (1.02, 2.32)	**1.58 (1.05, 2.39)**
Multigravidae (4th)	17.8	82.2	478	Ref	Ref
**Self-reported STI/RTI symptoms during current pregnancy**					
No	23.4	76.6	880	Ref	Ref
Yes	31.8	68.2	198	1.53 (1.09, 2.14)	**1.56 (1.11, 2.20)**
**Bacterial vaginosis**					
**Age group**					
< 20	44.6	55.4	202	1.10 (0.76, 1.57)	1.23 (0.85, 1.77)
20–29	52.7	47.3	571	1.53 (1.15, 2.02)	**1.58 (1.19, 2.10)**
30+	42.2	57.8	308	Ref	Ref
**HIV status**					
Negative	45.5	54.5	937	Ref	NA
Positive	66.4	33.6	143	2.37 (1.64, 3.44)	**2.41 (1.66, 3.51)**
**Syphilis**					
**Age group**					
< 20	2.0	98.0	204	Ref	Ref
20–29	8.6	91.4	568	4.72 (1.68, 13.25)	**3.96 (1.40, 11.20)**
30+	7.5	92.5	305	4.08 (1.39, 11.97)	**3.29(1.11, 9.74)**
**Number of lifetime sexual partners**					
≤ 2	5.7	94.3	792	Ref	Ref
≥ 3	11.3	88.7	275	2.11 (1.31, 3.41)	**1.82 (1.12, 2.98)**
**HIV status**					
Negative	5.8	94.2	938	Ref	Ref
Positive	15.8	84.2	139	3.08 (1.81, 5.24)	**2.56 (1.48, 4.41)**
**Any STI/RTI**					
**Age group**					
< 20	67.7	32.4	204	**1.56 (1.07, 2.25)**	**1.70 (1.16, 2.78)**
20–29	67.4	32.6	573	**1.54 (1.15, 2.04)**	**1.58 (1.18, 2.11)**
30+	57.3	42.6	307	**Ref**	**Ref**
**HIV status**					
Negative	62.8	37.2	940	**Ref**	**Ref**
Positive	76.9	23.3	143	**1.98 (1.31, 2.98)**	**2.10 (1.39, 3.19)**

^**¥**^For bacterial vaginosis the number included the intermediate and normal subgroups

*Adjusted for all the variables in the left-hand column of Supplemental Tables 1, 2, 3, 4, 5; The multivariate model only included factors that were significant at *P* < 0.05 using the likelihood ratio test. Ref = Reference group with the odds ratio set at 1

## Discussion

The prevalence of curable STIs among pregnant women is substantial throughout low- and middle-income countries [30]. Nearly two-thirds of all pregnant women in our cohort had a curable STI/RTI at antenatal booking. These findings are consistent with the pooled prevalence estimates of curable STIs/RTIs among pregnant women attending ANC services across East and Southern Africa [31]. The distribution of infection within this study group may have been affected by the large proportion of multigravidae, 60.7% (659 of 1085). This age structure may have made it more difficult to detect young age as a risk factor for chlamydia as reported elsewhere among pregnant [32] and non-pregnant women [33]. HIV-infected pregnant women aged less than 25 years were found to have higher odds of infection with chlamydia, gonorrhoea or trichomoniasis [34]. However, other studies have reported failure to identify any particular risk factors for chlamydial infection [35, 36] as in the current study. Risk factors associated with gonorrhoea were age related, i.e. being younger than age 30, similar to findings from Botswana and may suggest circulation of *Neisseria gonorrhoea* in younger networks of sexually active individuals [37].

Bacterial vaginosis was associated with HIV infection, a common observation among mothers with HIV [38, 39]. Syphilis prevalence was alarmingly high in our study, 7.1% overall. At the population level, syphilis infection tends to be more common among older women and, therefore, having a large proportion of multigravidae in our cohort may have skewed the prevalence of maternal syphilis upward. The reason for this may be related to the natural course of the disease. After initial ulcers of primary infection heal, syphilis often becomes asymptomatic, persisting untreated for years. In our cohort study, primi- and secundigravidae represented 36.8% of syphilis infections (28 of 76), whereas 63.2% (48 of 76) were multigravidae. Even given the age structure of our cohort, women of Nchelenge District may experience a disproportionate burden of maternal syphilis. In 2012, an estimated 2.4% of pregnant women in Zambia had syphilis, increasing marginally to 2.5% by 2016 [40]. In sub-Saharan Africa, 1.6% of pregnant women had syphilis in 2012, lowering slightly to 1.5% in 2016 [40].

Identification of risk factors for infection can play a role in the development of a profile for high risk groups for the purpose of directing the limited resources to where they are needed most. Development of a tool to identify women at high risk of infection can allow for either provision of presumptive treatment to high risk pregnant women or screening high risk pregnant women at ANC visits for STI/RTIs and provision of treatment for positive cases.

### Public health response

Syndromic management of curable STIs/RTIs clearly demonstrated limited utility given that only 10.2% of women with infections, apart from syphilis, received any treatment. Of the women with symptomatic curable STI/RTI, 43.9% received treatment according to national norms. Women were asked whether they were experiencing anything from a list of STI/RTI related symptoms. This created the basis of the classification of women to the symptomatic and asymptomatic group. However, responses to individual symptoms were not recorded. Consequently, it is not possible to draw conclusions about the appropriateness of the care that was administered.

Even under optimal circumstances, syndromic management is unlikely to perform much better given that 86% of the curable STIs/RTIs were asymptomatic in this setting. High prevalence of untreated STIs/RTIs means high prevalence of avoidable adverse pregnancy outcomes. The shortcomings of syndromic management in pregnancy have been documented [10, 32, 41], but public health responses have been lacking. Given that point of care (POC) tests *for C. trachomatis, N. gonorrhoea* and *T. vaginalis* are available [42], there is an urgent need to evaluate them in the antenatal setting as an alternative to syndromic management, particularly where risk factors may be used to guide testing [43]. Studies indicate that integration of POC diagnostic STI screening into first ANC among pregnant HIV-infected women is feasible [44, 45] and acceptable [44] although this has not been evaluated in mixed populations of HIV-infected and HIV-uninfected pregnant women. Due to lack of infrastructure and trained laboratory staff, conventional testing for STIs/RTIs is often unavailable in resource poor settings such as this study site. Until the necessary capacity is present, POC tests represent an important alternative for diagnosing chlamydia, gonorrhoea and trichomoniasis in such areas.

#### Chlamydia and gonorrhoea

Chlamydia and gonorrhoea POC tests merit investigation. Given that each test represents a fixed-cost, it would be useful to explore whether the use of POC tests among women who are at risk of chlamydia and gonorrhoea might be able to achieve similar reductions in adverse pregnancy outcomes attributable to these infections as universal screening and case management. In this study group, no specific risk factors for women who had chlamydial infection were identified. Consequently, universal POC testing should be considered in this setting first and foremost until a more targeted approach can be established based on risk profiles for chlamydial infection in this population. In the case of gonorrhoea, universal POC testing should be evaluated against an approach of targeted screening for pregnant women under 30 years of age, or those with HIV infection.

It would be important to establish differential diagnoses between chlamydia and gonorrhoea due of treatment implications, even though just three of 90 (3.3%) pregnant women in Nchelenge were co-infected. The WHO recommends treating chlamydia during pregnancy with a one-time 1 g dose of azithromycin. The same dose, however, may also hasten the emergence of untreatable gonorrhoea among women with co-infection [46]. Gonorrhoea in pregnancy, per WHO guidelines, should be treated with either ceftriaxone 250 mg by intramuscular injection plus azithromycin 1 g orally, or cefixime 400 mg orally plus azithromycin 1 g orally [47]. If differential diagnoses cannot be established, there may be reason to provide a curative dose for gonorrhoea which would clear co-infection and reduce opportunity for gonococcus to be exposed to azithromycin monotherapy. Screening and treatment of infected partners needs to be provided with such an approach. There is a convenient dual-cassette POC test available that can detect chlamydia and gonorrhoea with one biological sample [42], but cost is significant barrier to routine use.

#### Trichomoniasis and bacterial vaginosis

Universal screening and treatment for trichomoniasis and bacterial vaginosis would be an important improvement over syndromic management, particularly given how prevalent both are in this setting. Pregnant women diagnosed with either trichomoniasis or bacterial vaginosis could be given a single 2-g dose of metronidazole as recommended by the WHO [48]. Another option might be simply to provide metronidazole to all women at ANC booking. These two approaches need to be evaluated for their protective effect against adverse pregnancy outcomes and the cost per adverse outcome averted. Part of the comparison should include partner screening for trichomoniasis and appropriate case management.

#### Syphilis

Efforts must be redoubled to ensure syphilis POC tests are available to all women at ANC booking and that 2.4 MU benzathine penicillin G is in stock to treat cases. As with other curable STIs, partner screening and treatment is needed to prevent re-exposure from infected partners. In the case of syphilis, this is particularly important. Vertical transmission of syphilis has been reported in all stages and sub-stages of infection. However, congenital infection is more likely to occur and be most severe among pregnant women with first-stage syphilis (which includes primary, secondary and early-latent infections up to 2 years following exposure) than second-stage syphilis. Thus, if a woman is at risk of reinfection or resident in communities where syphilis prevalence is high, monthly quantitative titres are suggested from the third trimester to delivery [49].

This observational cross-sectional study is not without limitations. The prevalence and risk factors for curable STIs/RTIs we observed may not reflect all pregnant women in rural Zambia if there were differences between women who seek antenatal care and those who do not. Any difference, however, would not likely change the results substantially because ANC attendance (minimum one visit) in this part of Zambia was 94.6% [50], and the refusal rate in the study among eligible women was less than 1%. Another study limitation relates to the type of information required for comprehensive risk factor analyses. Some women, for example, may have been reluctant to provide accurate responses to questions related to sexual behaviour and practices. This is particularly the case given the increasing participation of male partners at the first ANC visit. In the current study, women were asked whether they were experiencing any STI/RTI symptoms. Responses, however, were not differentiated. Therefore, it was only possible to determine whether treatment was given to symptomatic women, but not if the treatment was appropriate. Another limitation is that information on alcohol or substance abuse was not collected. Finally, the study was under powered to detect risk factors with ORs < 1.5. Regardless, results from this observational cross-sectional study clearly show that the status quo is unacceptable and that new interventions are warranted. STI vaccines remains under development. Until they are ready to deploy, clinical trials are urgently needed across a range of epidemiological settings to identify the optimal mix of interventions that is efficacious, cost-effective, and superior to the syndromic management of STIs/RTIs in pregnancy.

## Conclusions

There was a high prevalence of curable STIs/RTIs in this population of pregnant women, a large proportion of which were not diagnosed and thus untreated. Syndromic management of curable STIS/RTIs is inadequate in this rural antenatal setting.

## Supplementary Information

**Additional file 1: Supplementary Table 1.** Chlamydia: Univariate and multivariate logistic regression analysis of potential risk factors of infection among pregnant women at antenatal care booking. **Supplementary Table 2.** Gonorrhoea: Univariate and multivariate logistic regression analysis of determinants of infection among pregnant women at antenatal care booking. **Supplementary Table 3.** Trichomoniasis: Univariate and multivariate logistic regression analysis of determinants of infection among pregnant women at antenatal care booking. **Supplementary Table 4.** Bacterial vaginosis: Univariate and multivariate logistic regression analysis of determinants of infection among pregnant women at antenatal care booking. **Supplementary Table 5.** Syphilis: Univariate and multivariate logistic regression analysis of determinants of infection among pregnant women at antenatal care booking. **Supplementary Table 6.** Any Sexually transmitted and reproductive tract infection: Univariate and multivariate logistic regression analysis of determinants of infection among pregnant women at antenatal care booking.

## Data Availability

The datasets used and/or analysed during the current study are available from the corresponding author on reasonable request.

## Abbreviations

ANC: Antenatal clinic
aOR: Adjusted Odds ratio
CI: Confidence interval
HIV: Human immunodeficiency virus
OR: Odds ratio
RPR: Rapid plasma reagin
STI/RTI: Sexually transmitted and reproductive tract infection
