# Supplementary material for: Assessment of syndromic management of curable sexually transmitted and reproductive tract infections among pregnant women: an observational cross-sectional study
Source: BMC Pregnancy Childbirth. 2021 Jan 30;21:98. doi: 10.1186/s12884-021-03573-3 (PMC7847014; doi:10.1186/s12884-021-03573-3)
Supplement: Supplementary file 1 — Additional file 1: Supplementary Table 1. Chlamydia: Univariate and multivariate logistic regression analysis of potential risk factors of infection among pregnant women at antenatal care booking. Supplementary Table 2. Gonorrhoea: Univariate and multivariate logistic regression analysis of determinants of infection among pregnant women at antenatal care booking. Supplementary Table 3. Trichomoniasis: Univariate and multivariate logistic regression analysis of determinants of infection among pregnant women at antenatal care booking. Supplementary Table 4. Bacterial vaginosis: Univariate and multivariate logistic regression analysis of determinants of infection among pregnant women at antenatal care booking. Supplementary Table 5. Syphilis: Univariate and multivariate logistic regression analysis of determinants of infection among pregnant women at antenatal care booking. Supplementary Table 6. Any Sexually transmitted and reproductive tract infection: Univariate and multivariate logistic regression analysis of determinants of infection among pregnant women at antenatal care booking. [file 12884_2021_3573_MOESM1_ESM.docx]

**Supplementary Table 1.** Chlamydia: Univariate and multivariate logistic regression analysis of potential risk factors of infection among pregnant women at antenatal care booking.

| **Potential risk factor** | **Chlamydia** | | **N** | **Crude odds ratio**  **(95% CI)** |
| --- | --- | --- | --- | --- |
|  | **% positive**  **(n = 56)** | **% negative**  **(n = 1028)** |  |  |
| **Age group** | | | | |
| <20 | 6.4 | 93.6 | 204 | Ref |
| 20 – 29 | 5.6 | 94.4 | 573 | 0.69 (0.38, 1.26) |
| 30+ | 3.6 | 96.4 | 307 | 0.48 (0.23, 1.03) |
| **Education** | | | | |
| Primary School or less | 4.5 | 95.5 | 600 | Ref |
| Junior secondary | 6.2 | 93.8 | 307 | 1.40 (0.77, 2.56) |
| Secondary School or higher | 5.7 | 94.4 | 177 | 1.27 (0.60-2.68) |
| **Marital status** | | | | |
| Single | 5.9 | 94.1 | 203 | Ref |
| Married | 5.0 | 95.0 | 873 | 0.84 (0.44, 1.63) |
| Separated /widowed/divorced | 0.0 | 100.0 | 8 | NA |
| **Pregnancy number** | | | | |
| Primigravidae | 28.6 | 23.8 | 261 | Ref |
| Secundigravidae | 21.4 | 14.9 | 165 | 1.20 (0.55, 2.60) |
| Multigravidae (3^rd^) | 12.5 | 16.8 | 180 | 0.62 (0.25, 1.54) |
| Multigravidae (4^th^) | 37.5 | 44.5 | 478 | 0.70 (0.36, 1.37) |
| **Number of sexual partners** | | | | |
| < 2 | 5.3 | 94.7 | 797 | Ref |
| > 3 | 5.1 | 94.9 | 277 | 0.96 (0.51, 1.78) |
| **Self-reported STI/RTI symptoms during current pregnancy** | | | | |
| No | 4.9 | 95.1 | 880 | Ref |
| Yes | 6.1 | 93.9 | 198 | 1.26 (0.65, 2.43) |
| **HIV status** | | | | |
| Negative | 4.9 | 95.1 | 940 | Ref |
| Positive | 7.0 | 93.0 | 143 | 1.46 (0.72, 2.97) |
| Missing | NK | NK | 1 | NA |
| Notes: 1. Diagnostic results for chlamydia were available for 1084 participants instead of 1085, the cervico-vaginal swab sample for one participant was missing; 2. The multivariate model only included factors that were significant at *P* < 0.05 using the likelihood ratio test.; NA = Not applicable; NK = Not known value; Ref = Reference group with the odds ratio set at 1. | | | | |

**Supplementary Table 2.** Gonorrhoea: Univariate and multivariate logistic regression analysis of determinants of infection among pregnant women at antenatal care booking.

| **Potential risk factor** | **Gonorrhoea** | | **N** | **Crude odds**  **ratio**  **(95% CI)** | **Adjusted odds ratio^*^**  **(95% CI)** | ***P*-value** |
| --- | --- | --- | --- | --- | --- | --- |
|  | **% positive**  **(n = 34)** | **% negative**  **(n = 1,050)** |  |  |  |  |
| **Age group** | | | | | |  |
| <20 | 3.9 | 96.1 | 204 | 4.14 (1.08, 15.78) | **5.01 (1.29, 19.44)** | **0.020** |
| 20 – 29 | 4.0 | 96.0 | 573 | 4.24 (1.26, 14.22) | **4.27 (1.32, 14.94)** | **0.016** |
| 30+ | 1.0 | 99.0 | 307 | Ref | Ref |  |
| **Education** | | | | | |  |
| Primary School or less | 2.0 | 98.0 | 600 | Ref | NA | - |
| Junior secondary | 3.9 | 96.1 | 307 | 1.99 (0.88, 4.49) | NA | - |
| Secondary School or higher | 5.6 | 94.4 | 177 | 2.93 (1.25, 6.91) | NA | - |
| **Marital status** | | | | | |  |
| Single | 2.0 | 98.0 | 203 | Ref | NA | - |
| Married | 3.4 | 96.6 | 873 | 1.77 (0.62, 5.08) | NA | - |
| Separated /widowed/divorced | 0.0 | 100.0 | 8 | NA | NA | - |
| **Pregnancy number** | | | | | |  |
| Primigravidae | 3.5 | 96.6 | 261 | Ref | NA | - |
| Secundigravidae | 4.9 | 95.2 | 165 | 1.42 (0.54, 3.77) | NA | - |
| Multigravidae (3^rd^) | 2.8 | 97.2 | 180 | 0.80 (0.26, 2.43) | NA | - |
| Multigravidae (4^th^) | 2.5 | 97.5 | 478 | 0.72 (0.30, 1.73) | NA | - |
| **Number of lifetime sexual partners** | | | | | |  |
| < 2 | 3.0 | 97.0 | 797 | Ref | NA | - |
| > 3 | 3.6 | 96.4 | 277 | 1.20 (0.56, 2.55) | NA | - |
| **Self-reported STI/RTI symptoms during current pregnancy** | | | | | |  |
| No | 2.7 | 97.3 | 880 | Ref | NA | - |
| Yes | 5.0 | 95.0 | 198 | 1.90 (0.89, 4.03) | NA | - |
| Missing | NK | NK | 6 | NA | NA | - |
| **HIV status** | | | | | |  |
| Negative | 2.7 | 97.3 | 940 | Ref | Ref | - |
| Positive | 6.3 | 93.7 | 143 | **2.46 (1.12, 5.38)** | **2.80 (1.25, 6.26)** | **0.012** |
| Missing | NK | NK | 1 | **NA** | NA | - |
| *Adjusted for all the variables in the column; Notes: 1. Diagnostic results for gonorrhoea were available for 1084 participants instead of 1085. The cervico-vaginal swab sample for one participant was missing; 2. The multivariate model only included factors that were significant at *P* < 0.05 using the likelihood ratio test. NK = Not known; NA = Not applicable; Ref = Reference group with the odds ratio set at 1. | | | | | | |

**Supplementary Table 3.** Trichomoniasis: Univariate and multivariate logistic regression analysis of determinants of infection among pregnant women at antenatal care booking.

| **Potential risk factor** | **Trichomoniasis** | | **N** | **Crude odds ratio**  **(95% CI)** | **Adjusted odds ratio**  **(95% CI)*** | ***P*-value** |
| --- | --- | --- | --- | --- | --- | --- |
|  | **% positive**  **(n = 269)** | **% negative**  **(n =815)** |  |  |  |  |
| **Age group** | | | | | | |
| <20 | 31.9 | 68.1 | 204 | 2.19 (1.45, 3.32) | NA | - |
| 20 – 29 | 26.2 | 73.8 | 573 | 1.66 (1.17, 2.35) | NA | - |
| 30+ | 17.6 | 82.4 | 307 | Ref | NA | - |
| **Education** | | | | | | |
| Primary School or less | 23.8 | 76.2 | 600 | Ref | NA | - |
| Junior secondary | 24.1 | 75.9 | 307 | 1.01 (0.74, 1.40) | NA | - |
| Secondary School or higher | 29.4 | 70.6 | 177 | 1.32 (0.91, 1.93) | NA | - |
| **Marital status** | | | | | | |
| Single | 34.0 | 66.0 | 203 | Ref | NA | - |
| Married | 22.7 | 77.3 | 873 | 0.56 (0.41, 0.79) | NA | - |
| Separated/widowed/divorced | 25.0 | 75.0 | 8 | 0.65 (0.13, 3.29) | NA | - |
| **Pregnancy number** | | | | | | |
| Primigravidae | 34.1 | 65.9 | 261 | 2.39 (1.69, 3.38) | **2.40 (1.69, 3.40)** | **< 0.001** |
| Secundigravidae | 30.3 | 69.7 | 165 | 2.01 (1.34, 3.02) | **2.06 (1.37, 3.10)** | **0.001** |
| Multigravidae (3^rd^) | 25.0 | 75.0 | 180 | 1.54 (1.02, 2.32) | **1.58 (1.05, 2.39)** | **0.03** |
| Multigravidae (4^th^) | 17.8 | 82.2 | 478 | Ref | Ref | - |
| **Self-reported STI/RTI symptoms during current pregnancy** | | | | | | |
| No | 23.4 | 76.6 | 880 | Ref | Ref | - |
| Yes | 31.8 | 68.2 | 198 | 1.53 (1.09, 2.14) | **1.56 (1.11, 2.20)** | **0.011** |
| **Number of sexual partners** | | | | | | |
| $\leq2$ | 23.5 | 76.5 | 797 | Ref | NA | - |
| $\geq3$ | 28.5 | 71.5 | 277 | 1.30 (0.95, 1.77) | NA | - |
| **HIV status** | | | | | | |
| Negative | 25.0 | 75.0 | 940 | Ref | NA | - |
| Positive | 23.8 | 76.2 | 143 | 0.94 (0.62, 1.41) | NA | - |
| *Adjusted for all the variables in the column; Note diagnostic results for trichomoniasis were available for 1084 participants instead of 1085. The cervico-vaginal swab sample for one participant was missing; The final multivariate model only included factors that were significant at *P* < 0.05 using the likelihood ratio test; Ref = Reference group with the odds ratios set at 1. | | | | | | |

**Supplementary Table 4.** Bacterial vaginosis: Univariate and multivariate logistic regression analysis of determinants of infection among pregnant women at antenatal care booking.

| **Potential risk factor** | **Bacterial vaginosis^¥^** | | **N** | **Crude odds**  **ratio**  **(95% CI)** | **Adjusted odds ratio**  **(95% CI)*** | ***P*-value** |
| --- | --- | --- | --- | --- | --- | --- |
|  | **% positive**  **(n = 521)** | **% negative**  **(n = 560)** |  |  |  |  |
| **Age group** | | | | | | |
| <20 | 44.6 | 55.5 | 202 | 1.10 (0.76, 1.57) | 1.23 (0.85, 1.77) | - |
| 20 – 29 | 52.7 | 47.3 | 571 | 1.53 (1.15, 2.02) | **1.58 (1.19, 2.10)** | **0.002** |
| 30+ | 42.2 | 57.8 | 308 | Ref | Ref | - |
| **Education** | | | | | | |
| Primary School or less | 45.2 | 54.9 | 598 | Ref | NA | - |
| Junior secondary | 51.8 | 48.2 | 305 | 1.31 (0.99, 1.72) | NA | - |
| Secondary School or higher | 52.3 | 47.8 | 178 | 1.33 (0.95, 1.86) | NA | - |
| **Marital status** | | | | | | |
| Single | 43.8 | 56.2 | 201 | Ref | NA | - |
| Married | 49.3 | 50.7 | 872 | 1.24 (0.92, 1.70) | NA | - |
| Separated/widowed/divorced | 37.5 | 62.5 | 8 | 0.77 (0.18, 3.31) | NA | - |
| **Pregnancy number** | | | | | | |
| Primigravidae | 48.7 | 51.4 | 259 | 1.12 (0.86, 1.58) | NA | - |
| Secundigravidae | 52.4 | 47.6 | 164 | 1.36 (0.95, 1.94) | NA | - |
| Multigravidae (3^rd^) | 52.8 | 47.2 | 180 | 1.38 (0.98, 1.94) | NA | - |
| Multigravidae (4^th^) | 44.8 | 55.3 | 478 | Ref | NA | - |
| **Number of life-time sexual partners** | | | | | | |
| $\leq2$ | 47.7 | 52.3 | 795 | Ref | NA | - |
| $\geq3$ | 50.0 | 50.0 | 276 | 1.10 (0.83, 1.44) | NA | - |
| **Self-reported STI/RTI symptoms during current pregnancy** | | | | | | |
| No | 47.6 | 52.5 | 879 | Ref | NA | - |
| Yes | 52.0 | 48.0 | 196 | 1.20 (0.88, 1.63) | NA | - |
| **HIV status** | | | | | | |
| Negative | 45.5 | 54.5 | 937 | Ref | NA | - |
| Positive | 66.4 | 33.6 | 143 | 2.37 (1.64, 3.44) | **2.41 (1.66, 3.51)** | **< 0.001** |
| **STIs^+^** | | | | | | |
| Negative | 46.1 | 54.0 | 708 | Ref | NA | - |
| Positive | 52.4 | 47.6 | 372 | 1.29 (1.00, 1.66) | NA | - |
| *Adjusted for all the variables in the column; ^+^ STIs included, syphilis, chlamydia, gonorrhoea and trichomoniasis.  **^¥^** Diagnostic results for bacterial vaginosis were available for 1081 participants instead of 1085. This was due to missing results. Three slides were poorly prepared and one was broken; The final multivariate model only included factors that were significant at *P* < 0.05 using the likelihood ratio test. Ref = Reference group with the odds ratios set at 1. | | | | | | |

**Supplementary Table 5.** Syphilis: Univariate and multivariate logistic regression analysis of determinants of infection among pregnant women at antenatal care booking.

| **Potential risk factor** | **Syphilis** | | **N** | **Crude odds**  **ratio**  **(95% CI)** | **Adjusted odds ratio***  **(95% CI)** | ***P*-value** |
| --- | --- | --- | --- | --- | --- | --- |
|  | **% positive**  **(n = 76)** | **% negative**  **(n = 1,001)** |  |  |  |  |
| **Age group** | | | | | | |
| <20 | 2.0 | 98.0 | 204 | Ref | Ref | - |
| 20 – 29 | 8.6 | 91.4 | 568 | 4.72 (1.68, 13.25) | **3.96 (1.40, 11.20)** | **0.009** |
| 30+ | 7.5 | 92.5 | 305 | 4.08 (1.39, 11.97) | **3.29(1.11, 9.74)** | **0.032** |
| **Education** | | | | | | |
| Primary School or less | 8.4 | 91.6 | 594 | Ref | NA | - |
| Junior secondary | 4.9 | 95.1 | 305 | 0.56 (0.31, 1.02) | NA | - |
| Secondary School or higher | 6.2 | 87.5 | 178 | 0.72 (0.36, 1.41) | NA | - |
| **Marital status** | | | | | | |
| Single | 5.5 | 94.6 | 202 | Ref | NA | - |
| Married | 7.4 | 92.6 | 867 | 1.38 (0.71, 2.67) | NA | - |
| Separated /widowed/divorced | 12.5 | 87.5 | 8 | 2.48 (0.28, 21.98) | NA | - |
| **Pregnancy number** | | | | | | |
| Primigravidae | 5.0 | 95.0 | 260 | Ref | NA | - |
| Secundigravidae | 9.1 | 90.9 | 165 | 1.90 (0.88, 4.10) | NA | - |
| Multigravidae (3^rd^) | 6.7 | 93.3 | 178 | 1.37 (0.61, 3.08) | NA | - |
| Multigravidae (4^th^) | 7.6 | 92.9 | 474 | 1.56 (0.81, 3.00) | NA | - |
| **Number of lifetime sexual partners** | | | | | | |
| < 2 | 5.7 | 94.3 | 792 | Ref | Ref | - |
| > 3 | 11.3 | 88.7 | 275 | 2.11 (1.31, 3.41) | **1.82 (1.12, 2.98)** | **0.017** |
| **Self-reported STI/RTI symptoms during current pregnancy** | | | | | | |
| No | 6.8 | 93.3 | 874 | Ref | NA | - |
| Yes | 8.1 | 91.9 | 197 | 1.22 (0.69, 2.17) | NA | - |
| Missing | NK | NK | 6 | NA | NA | - |
| **HIV status** | | | | | | |
| Negative | 5.8 | 94.2 | 938 | Ref | Ref | - |
| Positive | 15.8 | 84.2 | 139 | 3.08 (1.81, 5.24) | **2.56 (1.48, 4.41)** | **0.001** |
| **Bacterial vaginosis** | | | | | | |
| Negative | 5.3 | 94.8 | 552 | Ref | NA | - |
| Positive | 9.0 | 91.0 | 521 | 1.79 (1.11, 2.89) | NA | - |
| Missing | NK | NK | 4 | NA | NA | - |
| *Adjusted for all the variables in the column; Note that diagnostic results for syphilis were available for 1077 participants instead of 1084. This was due to missing results. The multivariate model only included factors that were significant at P < 0.05 using the likelihood ratio test; NA = Not applicable; NK = Not known; Ref = Reference group with the odds ratios set at 1. | | | | | | |

**Supplementary Table 6.** Any Sexually transmitted and reproductive tract infection: Univariate and multivariate logistic regression analysis of determinants of infection among pregnant women at antenatal care booking

|  | **Any STI/RTI** | | **N** | **Crude odds**  **ratio**  **(95% CI)** | **Adjusted odds ratio^*^**  **(95% CI)** | ***P*-value** |
| --- | --- | --- | --- | --- | --- | --- |
|  | **% positive**  **(n = 700)** | **% negative**  **(n = 384)** |  |  |  |  |
| **Age group** | | | | | | |
| <20 | 67.7 | 32.4 | 204 | **1.56 (1.07, 2.25)** | **1.70 (1.16-2.78)** | **0.001** |
| 20 – 29 | 67.4 | 32.6 | 573 | **1.54 (1.15, 2.04)** | **1.58 (1.18-2.11)** | **0.001** |
| 30+ | 57.3 | 42.6 | 307 | **Ref** | **Ref** | - |
| **Education** | | | | | | |
| Primary School or less | 61.8 | 32.2 | 600 | Ref | NA | - |
| Junior secondary | 66.8 | 33.2 | 307 | 1.24 (0.93, 1.66) | NA | - |
| Secondary School or higher | 70.1 | 29.9 | 177 | 1.44 (1.01, 2.07) | NA | - |
| **Marital status** | | | | | | |
| Single | 66.5 | 33.5 | 203 | Ref | NA | - |
| Married | 64.2 | 35.9 | 873 | 0.90 (0.65, 1.24) | NA | - |
| Separated /widowed/divorced | 62.5 | 37.5 | 8 | 0.83 (0.19, 3.61) | NA | - |
| **Pregnancy number** | | | | | | |
| Primigravidae | 68.2 | 31.8 | 261 | Ref | NA | - |
| Secundigravidae | 70.3 | 29.7 | 165 | 1.10 (0.72-1.69) | NA | - |
| Multigravidae (3^rd^) | 67.8 | 32.2 | 180 | 0.98 (0.65-1.47) | NA | - |
| Multigravidae (4^th^) | 59.4 | 40.6 | 478 | 0.68 (0.49-0.94) | NA | - |
| **Number of lifetime sexual partners** | | | | | | |
| < 2 | 64.0 | 36.0 | 797 | Ref | NA | - |
| > 3 | 66.4 | 33.6 | 277 | 0.11 (0.83, 1.49) | NA | - |
| **Self-reported STI/RTI symptoms during current pregnancy** | | | | | | |
| No | 63.6 | 36.4 | 880 | Ref | NA | - |
| Yes | 69.7 | 30.3 | 198 | 1.31 (0.94, 1.83) | NA | - |
| Missing | NK | NK | 6 | NA | NA | - |
| **HIV status** | | | | | | |
| Negative | 62.8 | 37.2 | 940 | **Ref** | **Ref** | - |
| Positive | 76.9 | 23.3 | 143 | **1.98 (1.31, 2.98)** | **2.10 (1.39, 3.19)** | **0.030** |
| Missing | NK | NK | 1 | **NA** | **NA** | - |
| *Adjusted for all the variables in the column; Notes: 1. Diagnostic results for any STI/RTI were available for 1084 participants instead of 1085. The cervico-vaginal swab sample for one participant was missing; 2. The multivariate model only included factors that were significant at *P* < 0.05 using the likelihood ratio test. NK = Not known; NA = Not applicable; Ref = Reference group with the odds ratios set at 1. | | | | | | |
